# Silicosis and Silica-Induced Autoimmunity in the Diversity Outbred Mouse

**DOI:** 10.3389/fimmu.2018.00874

**Published:** 2018-04-26

**Authors:** Jessica M. Mayeux, Gabriela M. Escalante, Joseph M. Christy, Rahul D. Pawar, Dwight H. Kono, Kenneth M. Pollard

**Affiliations:** ^1^Department of Molecular Medicine, The Scripps Research Institute, La Jolla, CA, United States; ^2^Department of Immunology and Microbiology, The Scripps Research Institute, La Jolla, CA, United States

**Keywords:** silica, diversity outbred, autoimmunity, silicosis, autoantibody, inflammation, lung, environmental autoimmunity

## Abstract

Epidemiological studies have confidently linked occupational crystalline silica exposure to autoimmunity, but pathogenic mechanisms and role of genetic predisposition remain poorly defined. Although studies of single inbred strains have yielded insights, understanding the relationships between lung pathology, silica-induced autoimmunity, and genetic predisposition will require examination of a broad spectrum of responses and susceptibilities. We defined the characteristics of silicosis and autoimmunity and their relationships using the genetically heterogeneous diversity outbred (DO) mouse population and determined the suitability of this model for investigating silica-induced autoimmunity. Clinically relevant lung and autoimmune phenotypes were assessed 12 weeks after a transoral dose of 0, 5, or 10 mg crystalline silica in large cohorts of DO mice. Data were further analyzed for correlations, hierarchical clustering, and sex effects. DO mice exhibited a wide range of responses to silica, including mild to severe silicosis and importantly silica-induced systemic autoimmunity. Strikingly, about half of PBS controls were anti-nuclear antibodies (ANA) positive, however, few had disease-associated specificities, whereas most ANAs in silica-exposed mice showed anti-ENA5 reactivity. Correlation and hierarchical clustering showed close association of silicosis, lung biomarkers, and anti-ENA5, while other autoimmune characteristics, such as ANA and glomerulonephritis, clustered separately. Silica-exposed males had more lung inflammation, bronchoalveolar lavage fluid cells, IL-6, and autoantibodies. DO mice are susceptible to both silicosis and silica-induced autoimmunity and show substantial individual variations reflecting their genetic diverseness and the importance of predisposition particularly for autoimmunity. This model provides a new tool for deciphering the relationship between silica exposure, genes, and disease.

## Introduction

Crystalline silica is an abundant mineral found in rock, sand, and soil, and exposure is an environmental or occupational hazard in construction, mining, and other dusty trades (1, 2). Silicosis results from the inhalation and deposition of respirable crystalline silica in the lungs, which causes a range of pathologies, including inflammation, tissue damage, necrosis, and fibrosis (1–3). Silica exposure is also associated with autoimmunity [systemic lupus erythematosus (SLE), rheumatoid arthritis (RA), and scleroderma] in humans, however, little is known of the mechanisms responsible (2, 4–11). Although these diseases occur predominantly in females (12), silica-induced autoimmunity is most often described following occupational exposure in males (8). While several human studies have found an association between the intensity of silica exposure with autoimmune disease and autoantibodies, silica-induced autoantibody production can occur without significant lung damage (13–15). Silica exposure results in more pronounced lung responses and exacerbates autoimmunity in susceptible mice; however, the limited animal studies of silica-induced autoimmunity have revealed little regarding the mechanisms involved (9, 16–19). This paucity of information is a significant barrier to furthering our understanding of how silica-mediated pathology leads to autoimmunity.

Genetic predisposition and environmental exposure to silica likely interact to drive susceptibility or resistance to silicosis and silica-induced autoimmunity. Disease features associated with silica exposure occur with variable frequency in humans suggesting a significant gene–environment interaction in both silicosis and silica-induced autoimmunity (2). A similar spectrum of disease features are found in murine silicosis, which exhibits a clear dose response (16, 20), however variation among inbred mouse strains suggests a strong genetic component. Although this variability of disease phenotypes is observed when multiple inbred mouse strains are exposed to silica, it is not found in any single inbred mouse strain because the restricted genetic content recapitulates only some features of disease (9, 16, 20). These animal studies suggest that many of the pathological features described in human silicosis and silica-induced autoimmunity are heavily influenced by genetic background. However, studies to elucidate the genetic contribution to disease have been hampered by the lack of animal models that exemplify the genetic heterogeneity of human populations.

One approach to broaden the genetic heterogeneity in murine studies is to use outbred strains, such as the diversity outbred (DO) mouse (21–24). The DO mouse is a heterogeneous stock derived from eight founder strains (A/J, C57BL/6J, 129S1/SvImJ, NOD/ShiLtJ, NZO/HiLtJ, CAST/EiJ, PWK/PhJ, and WSB/EiJ) that is maintained by randomized breeding. The DO exhibits significant phenotypic variability because it captures the same set of allelic variants as the eight founder strains (25) and is, therefore, well suited to model the range of immunological responses that follow exposure to environmental agents known to induce disease in humans (26, 27). The DO mouse also provides a powerful tool for genome-wide association studies that could better model the genetic heterogeneity of human populations than inbred mice for the study of silicosis and silica-induced autoimmunity.

In this study, we sought to determine if DO mice could be used to model the diversity of immunological and pathological changes found in human silicosis and silica-induced autoimmunity. Here, we identify biomarkers of silica-induced lung pathology and autoimmunity and describe the range of responses to silica in the DO mouse establishing it as a model of population-based silicosis and silica-induced autoimmunity. Biomarkers associated with human pulmonary silicosis, bronchoalveolar lavage fluid (BALF) cell numbers and protein, TGF-β, TNF-α, and lactate dehydrogenase (LDH) activity, were increased in silica-exposed mice. Silica-induced autoimmunity, including serum IgM, IgG, anti-nuclear antibodies (ANA), and anti-ENA (RNP and Sm) specificity were increased with silica exposure along with lupus-like glomerulonephritis. Significantly, our findings also suggest that expression of some features of autoimmunity do not closely reflect silica-induced lung pathology, and that male mice can display more severe parameters of both silicosis and autoimmunity. Finally, these results provide a new model to study the effects of genetic diversity on the pathogenesis of silicosis and silica-induced autoimmunity.

## Materials and Methods

### Mice

Male and female DO mice (J:DO, Stock No: 009376) were purchased from Jackson Laboratory (Bar Harbor, ME, USA) and have been described previously (22). Handling and maintenance were performed under specific pathogen-free conditions at TSRI’s Animal Facility. Experiments were carried out with 8- to 10-week-old animals. Animal rooms were kept at 68–72°F and 60–70% humidity, with a 12/12 h light–dark cycle, and sterilized cages were replaced each week with fresh water and food (autoclaved standard grain diet 7012, Teklad, Envigo, Madison, WI, USA) to which the mice had access *ad libitum*. Behavioral enrichments, such as paper breeder huts, goat chow, and nylabones, were provided as needed. All procedures were approved by The Scripps Research Institute Institutional Animal Care and Use Committee (IACUC).

### Exposure to Crystalline Silica

Mice were exposed to a single dose of crystalline silica (Min-U-Sil-5, average particle size 1.5–2 µm; U.S. Silica Company, Frederick, MD, USA) by transoral (or oropharyngeal) instillation in a volume of 25 μl of PBS under isoflurane anesthesia. Briefly, mice were anesthetized and the tongue pulled forward and to the side (to block the swallow reflex) so that the silica solution is delivered into the back of the throat and aspirated into the lungs (28). A total of 210 8- to 10-week-old mice were exposed to crystalline silica, of which 90 received 5 mg and 120 received 10 mg. These doses were chosen based on calculations of human lifetime exposure to respirable crystalline silica and an equivalent exposure for mice, and represent approximately 60–120% of a human lifetime exposure at the recommended NIOSH exposure limit (19). A single instillation was used because of evidence of an association between the intensity of silica exposure and autoimmunity (8). A control group of 70 mice received PBS alone. Each treatment group consisted of equal numbers of male and female mice. Silica was acid washed in 1 M HCl at 100^°^C for 2 h and then autoclaved for 1 h at 121^°^C and allowed to dry (29). Immediately prior to use silica was disbursed by sonication. Use of crystalline silica was approved by The Scripps Research Institute Department of Environmental Health and Safety.

### Bronchoalveolar Lavage and Lung Histology

Mice were sacrificed at 12 weeks post-exposure and BALF was collected. Total cell counts were determined using a Countess II FL Automated Cell Counter (Thermo Fisher Scientific, Waltham, MA, USA) before BALF was stored at −80°C. Lungs were excised and fixed for 24 h in zinc formalin. Paraffin-embedding, sectioning (5 µm), and staining (hematoxylin and eosin and trichrome) were done by TSRI’s Histology Core. Formalin fixed paraffin-embedded slides were scanned and images stored on Digital Image Hub (Slidepath, Dublin, Ireland). Lungs were scored under blinded conditions. The four right lobes and the single left lung was scored for the percent of the lobe affected by alveolitis (maximum score of 500) as well as peribronchitis and perivasculitis (maximum score of 500). A total lung score (TLS) (maximum score of 1,000) was determined by combining the amount of alveolitis, peribronchitis, and perivasculitis. The presence of silicosis was defined as a TLS greater than the mean plus 2 SDs of the PBS controls (9.6).

### Immunofluorescence and ELISAs for Serum Immunoglobulins and Autoantibodies

Blood was collected from the retro-orbital sinus pre-silica exposure and at 4, 8, and 12 weeks post-exposure. Serum ANA were detected as previously described (30) using a 1:40 dilution of serum on Hep2 ANA slides as an initial screen (Innova Diagnostics, San Diego, CA, USA). Bound antibody was detected with goat anti-mouse IgG Alexa Fluor 488 (Life Technologies, Carlsbad, CA, USA). Slides were mounted using Vectashield Mounting Medium containing DAPI (Vectorlabs, Burlingame, CA, USA), and observed using an Olympus BH2 microscope. Digital images of ANA patterns were captured using a LEICA DFC 365 FX camera and analyzed using Leica Application Suite AF software (Leica Microsystems, Buffalo Grove, IL, USA).

Total serum IgG and IgM were measured by ELISA (30) as specified by the manufacturer (Immunology Consultants Laboratory, Portland, OR). To measure autoantibodies in the serum, ENA5 (designed for the detection of anti-Sm, -RNP, -SS-A (60 and 52 kDa), -SS-B, and -Scl-70 antibodies), anti-chromatin, anti-dsDNA, anti-SSA, anti-SSB, anti-RNP, and anti-Sm ELISA kits (QUANTA Lite ELISA Inova Diagnostics, San Diego, CA, USA) were modified to detect murine samples using a goat-anti mouse IgG HRP antibody (Thermo Fisher #62-6520). As calibrated mouse IgG of defined ANA specificities are not available the determination of antibody units in DO mice sera was achieved using human negative, low positive, and high positive controls and anti-human IgG HRP as described by the manufacturer (Innova Diagnostics). The units for each sample were calculated by dividing the average OD of the DO mouse sample by the average OD of the human Low Positive. The result was multiplied by the number of units assigned (by the manufacturer) to the human Low Positive (25 Units). Serum from MRL-*Fas^Lpr^* mice was used as positive controls for reactivity of mouse sera for all ELISAs and a murine monoclonal antibody against chromatin, MoAb 2–3 (courtesy of Dr. Marc Monestier, Temple University, Philadelphia, PA, USA) was additionally used for the anti-chromatin ELISA. For the measurement of IgM RF, a sandwich ELISA was performed by coating IgG1κ (BD Pharmingen, San Diego, CA, USA) at 1 μg/ml onto NUNC plates (Thermo Fisher Scientific) and blocked with PBS containing 1 mg gelatin/ml. Serum was diluted 1:200, and bound RF detected with goat anti-mouse IgM HRP (Invitrogen, Carlsbad, CA, USA) at 1:1,000.

### ELISA for Biomarkers in BALF

TNF-α (BioLegend, San Diego, CA, USA), IL-6 (BioLegend, San Diego, CA, USA), and activated TGF-β (R&D Systems, Minneapolis, MN, USA) were determined by ELISA according to the manufacturer’s instructions. LDH activity was measured using a colorimetric assay (Sigma-Aldrich, St. Louis, MO, USA). Protein in BALF was determined using the Pierce BCA Protein Assay Kit (Thermo Fisher Scientific, Waltham, MA, USA).

### Renal Pathology

Kidneys were fixed for 24 h in zinc formalin then paraffin sections (5 µm) were stained with Periodic Acid–Schiff (PAS). Slide images were stored on Digital Image Hub (Slidepath, Dublin, Ireland). Glomerulonephritis was scored on a 0–4 scale under blinded conditions (31). Proteinuria was determined using Roche Diagnostics Chemstrip 2 GP Urine Test Strips (Fisher Scientific).

### Statistical Analysis

Unless otherwise noted, data are expressed as mean and SE. Statistical analysis was performed with GraphPad Software V6 (San Diego, CA, USA). Comparisons were performed using Kruskal–Wallis test or two-way ANOVA with Sidak’s multiple comparisons. Correlations were calculated using pairwise deletion and reported in Spearman’s correlation coefficient (*r_s_*). Hierarchical Cluster Analysis was done using Cluster 3.0^1^ and Java TreeView^2^ using a relative change value for each phenotype. Briefly, relative change values were calculated using the equation: $\frac{s-s_{\text{avg}}}{s_{\text{avg}}}$, *s* = sample data point and *s*_avg_ = the average of the combined treatment samples (0, 5, and 10 mg). A one-dimensional self-organizing map was constructed along each axis (*x*-axis = samples, 20,000 iterations; *y*-axis = phenotypes, 100,000 iterations) followed by binary, agglomerative, hierarchical clustering using the Spearman Rank Correlation similarity metric, and a complete linkage clustering method. *P* < 0.05 was considered significant.

## Results

### Silica Exposure and Lung Pathology

**Figure 1:**
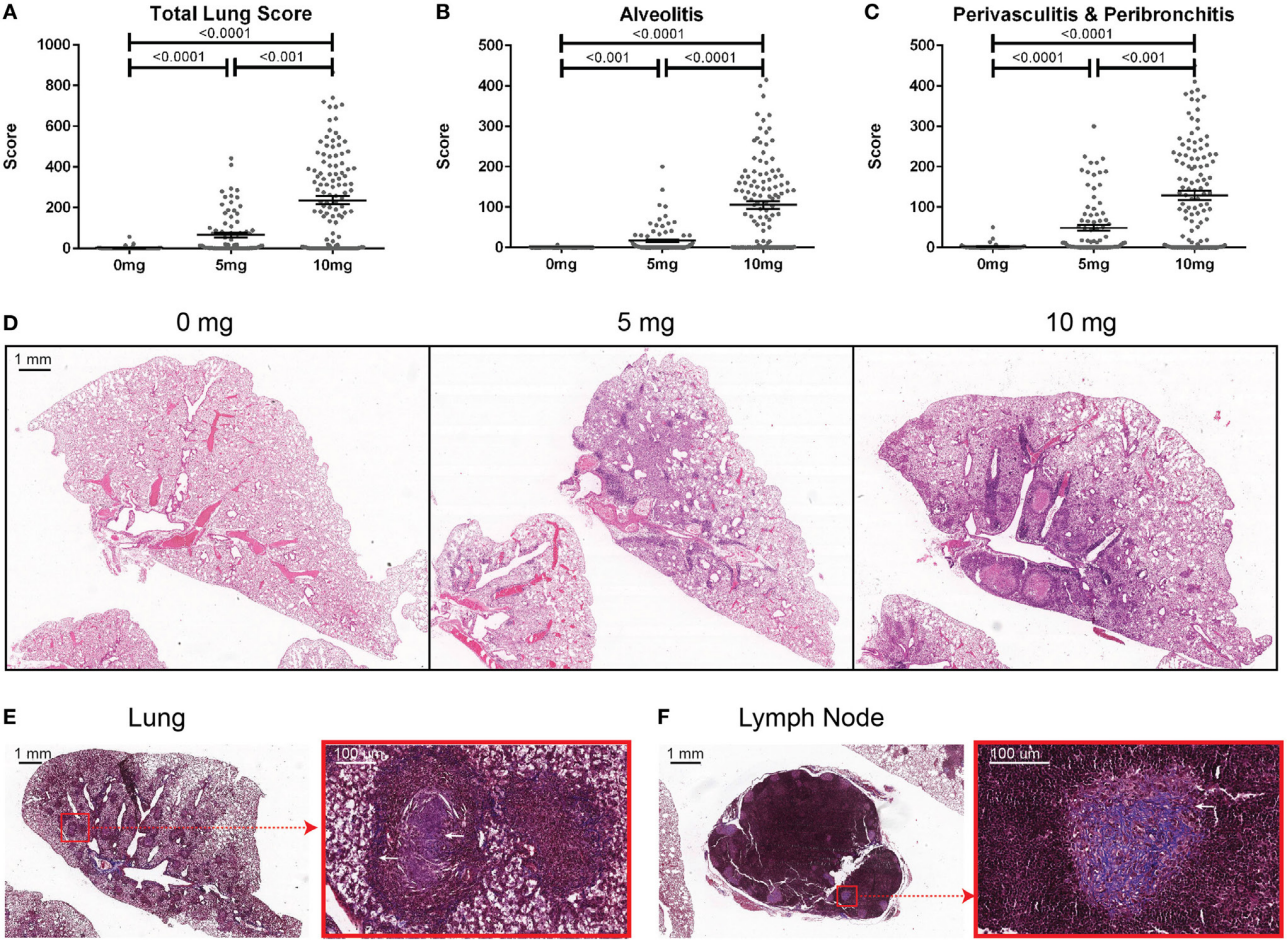
Indices of silica-induced lung pathology in diversity outbred mice. Mice were exposed transorally to 0 mg (*n* = 70), 5 mg (*n* = 90), or 10 mg (*n* = 120) of crystalline silica in PBS for 12 weeks before H&E stained lung sections were reviewed and scored for **(A)** total lung score, **(B)** alveolitis, and **(C)** peribronchitis and perivasculitis. Images of slides with **(D)** H&E staining of the left lung lobe from mice exposed to 0 (left), 5 (middle), and 10 mg (right) of crystalline silica in PBS. Trichrome staining of the **(E)** left lung lobe and **(F)** tracheobronchial lymph node with white arrows indicating granulomas containing lattice-like fibrous connective tissue (inset with red outline).

To determine the association of silicosis with autoimmunity, mice were given a single exposure to 0, 5, or 10 mg of crystalline silica and analyzed after 12 weeks. Silica exposure was well tolerated with no evidence of increased morbidity or any significant changes to whole body weight. Five animals died post exposure. Of these, two were PBS animals that died with mortal fighting-related wounds and three were silica-exposed animals with no fighting-related wounds, but all had high ANA positivity. One silica-treated animal exhibited extreme morbidity and edema during the exposure period and was sacrificed early. Mice exposed to either dose of silica developed prominent alveolar, peribronchial, and perivascular inflammatory cell infiltrates containing varying amounts of lymphocytes, macrophages, and neutrophils. Accordingly, the total lung, alveolitis, and perivasculitis/bronchitis pathology scores in silica-exposed mice were much greater than unexposed controls and exhibited a strong dose response (Figures 1A–D). Furthermore, there was a wide range of responses to the silica consistent with the genetic diversity of DO mice.

Several pathologic findings occurred in only subsets of silica-exposed DO mice, including fibrosis, granulomas, and enlarged tracheobronchial lymph nodes. Fibrosis was seen in both the lungs and the draining tracheobronchial lymph nodes of silica-exposed mice primarily within granulomas (Figures 1E,F). Tracheobronchial lymph node hypertrophy was not observed in PBS mice, but was observed in 23% of mice receiving 10 mg silica (*p* = 0.001). The frequency and size of granulomas varied widely within each dosage group, but was much more frequent at the higher dose.

### BALF Characteristics and Biomarkers of Silicosis

**Figure 2:**
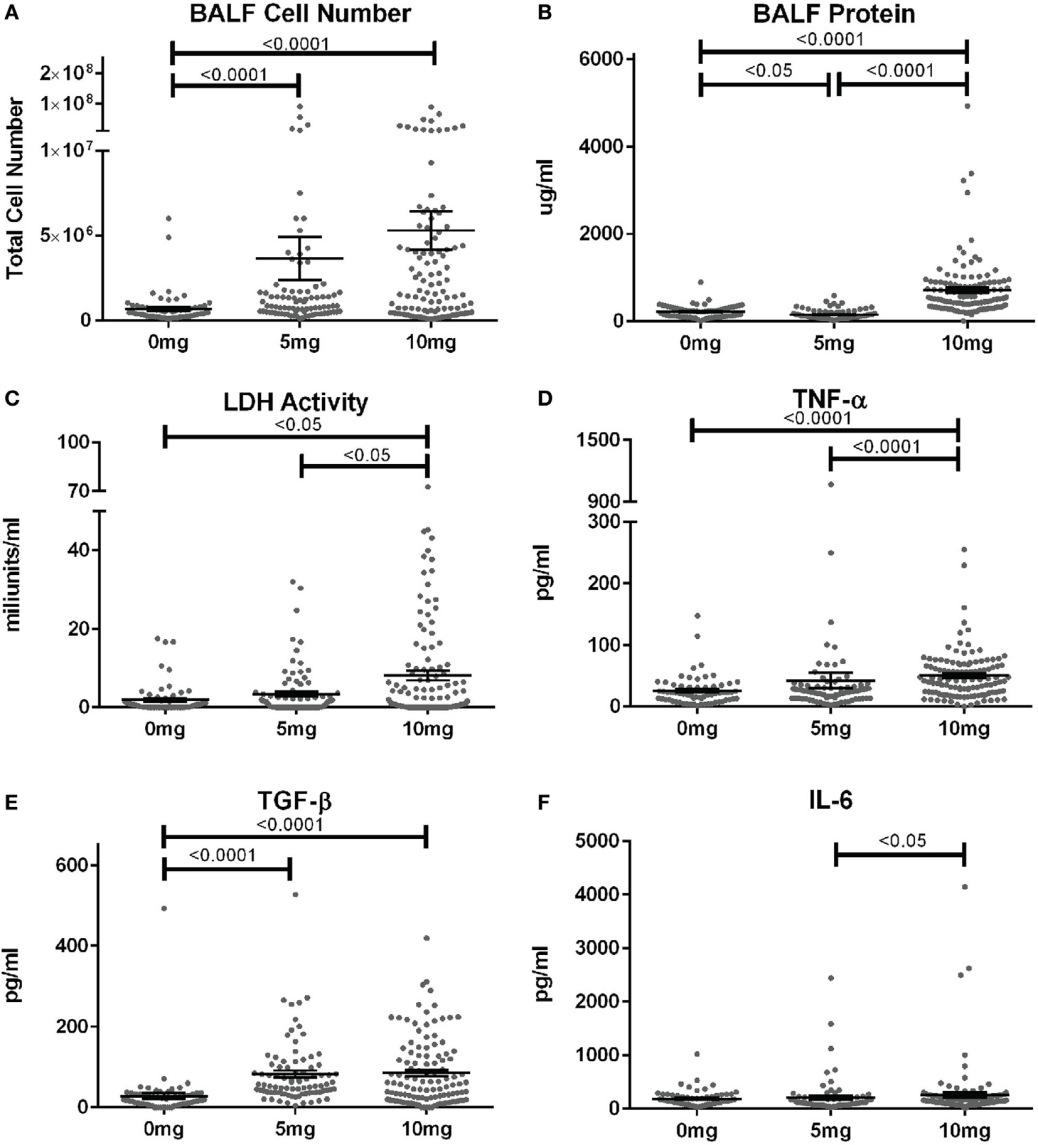
Biomarkers of inflammation and fibrosis in bronchial alveolar lavage fluid (BALF) of silica-exposed diversity outbred mice. Mice were exposed transorally to crystalline silica in PBS (see Figure 1) before BALF was collected for the measurement of **(A)** BALF cell number, **(B)** BALF protein levels, **(C)** lactate dehydrogenase activity, **(D)** TNF-α, **(E)** TGF-β, and **(F)** IL-6.

When corresponding BALF was examined, mean cell numbers and the percent of mice with elevated cell counts (greater than the mean + 2SD of PBS group) were substantially elevated in mice given 5 or 10 mg silica compared to PBS (Figure 2A; Table S1 in Supplementary Material). Furthermore, while the mean cell number was higher for the 10 versus 5 mg group, this did not reach statistical significance. Contrastingly, mean BALF protein levels and percent of mice with elevated protein levels were only increased in mice exposed to 10 mg of silica and unexpectedly mice receiving 5 mg of silica had slightly lower BALF protein levels than controls (Figure 2B). Thus, lower amounts of silica are sufficient to stimulate BALF cell increases, while higher doses are required to increase BALF protein levels.

We next determined whether BALF biomarkers of human silicosis (32) were also represented in silica-exposed DO mice. Indeed, mean levels of LDH activity, TNF-α, and TGF-β, but not IL-6 were elevated in mice exposed to silica (Figures 2C–F; Table S1 in Supplementary Material). Mean LDH activity increased with dose of silica, but only reached statistical significance with the 10 mg dose. Likewise, the percent of mice with elevated LDH activity was significantly higher in the 10 mg group. Similar findings were observed for TNF-α, however, the increases in mean values were only modest. In contrast, mean TGF-β levels were substantially higher in both silica dose groups. Taken together, our findings show that three of four reported BALF biomarkers for human pulmonary silicosis were elevated in a significant percentage of silica-exposed DO mice particularly at the higher exposure.

### Autoimmunity

**Figure 3:**
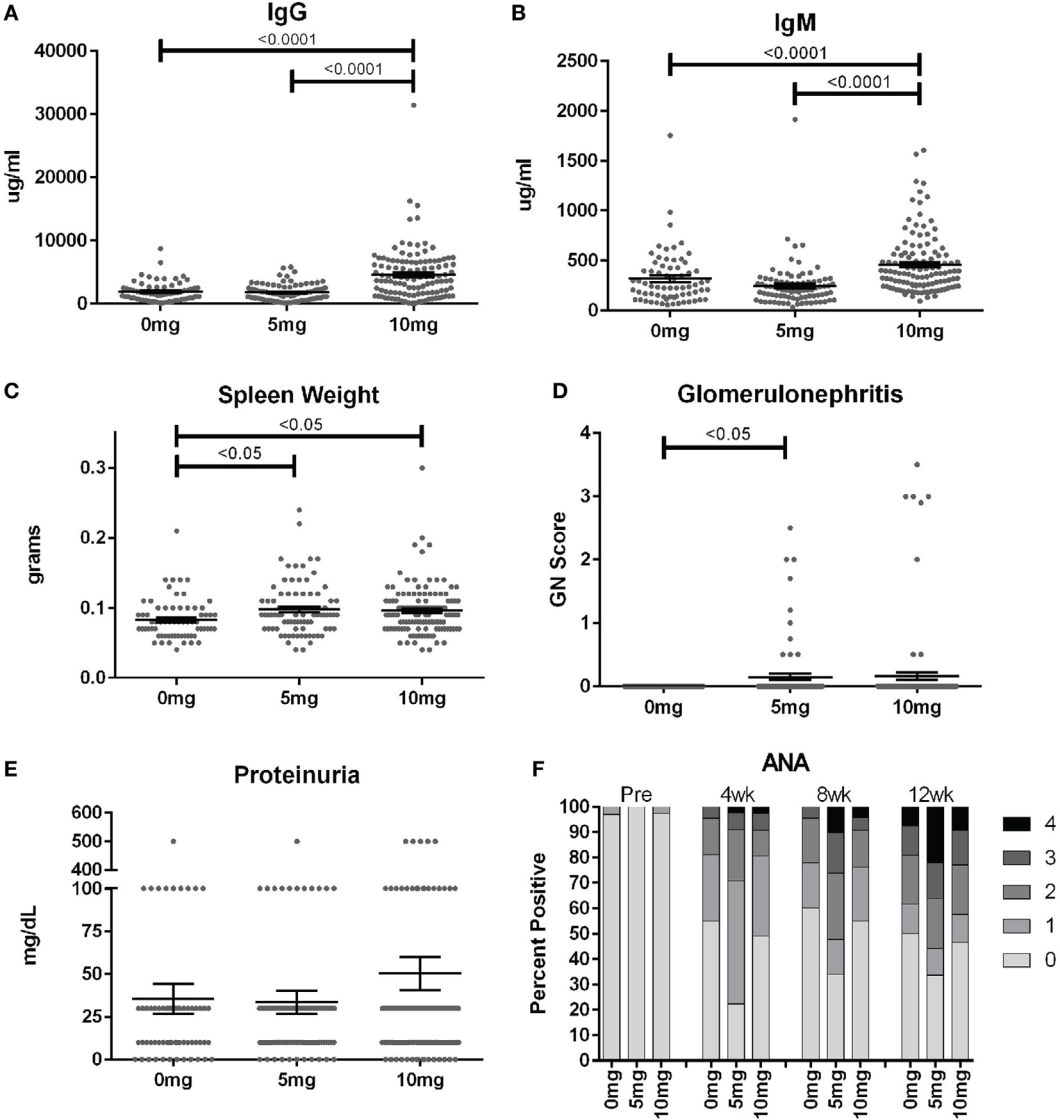
Indices of silica-induced autoimmunity in diversity outbred mice. Mice were exposed transorally to crystalline silica in PBS (see Figure 1) before blood was collected for determination of serum **(A)** IgG and **(B)** IgM and **(C)** spleen weight. **(D)** Glomerulonephritis was determined by reviewing and scoring Periodic Acid–Schiff-stained kidney sections and **(E)** proteinuria was measured in the urine using Chemstrips. **(F)** Blood was collected prior to exposure and every 4 weeks thereafter for determination of serum anti-nuclear antibodies by immunofluorescence.

To assess the frequency of systemic autoimmunity induced by silica exposure in the DO population, we examined immunoglobulin and autoantibody levels, spleen size, and kidney pathology. Mean serum IgM and IgG levels were significantly elevated 12 weeks after exposure in mice given 10, but not 5 mg of silica when compared to PBS controls, consistent with greater activation of the adaptive immune response with higher silica exposure (Figures 3A,B; Table S2 in Supplementary Material). Serial ANAs were measured prior to and every 4 weeks after PBS or silica exposure. Strikingly, 45% of mice in the PBS group became positive for ANA (≥1+) at 4 weeks (12–14 weeks of age) despite only two mice being ANA positive (both 1+) at the start of the experiment (Figure 3F; Table S2 in Supplementary Material). By 12 weeks, a similar 50% of PBS mice were ANA positive, however, the average ANA score was increased compared to 4 weeks (*p* < 0.0001). The 5 mg-exposed mice were 77% ANA positive at 4 weeks and 66% at 12 weeks. Mean ANA scores also increased over the course of the 12 weeks exposure (*p* < 0.0001) and were significantly higher than the PBS group (*p* = 0.006). In contrast, mice exposed to 10 mg of silica exhibited ANA positivity (50 and 53%, 4 and 12 weeks) and scores that were not significantly different from PBS controls.

**Figure 4:**
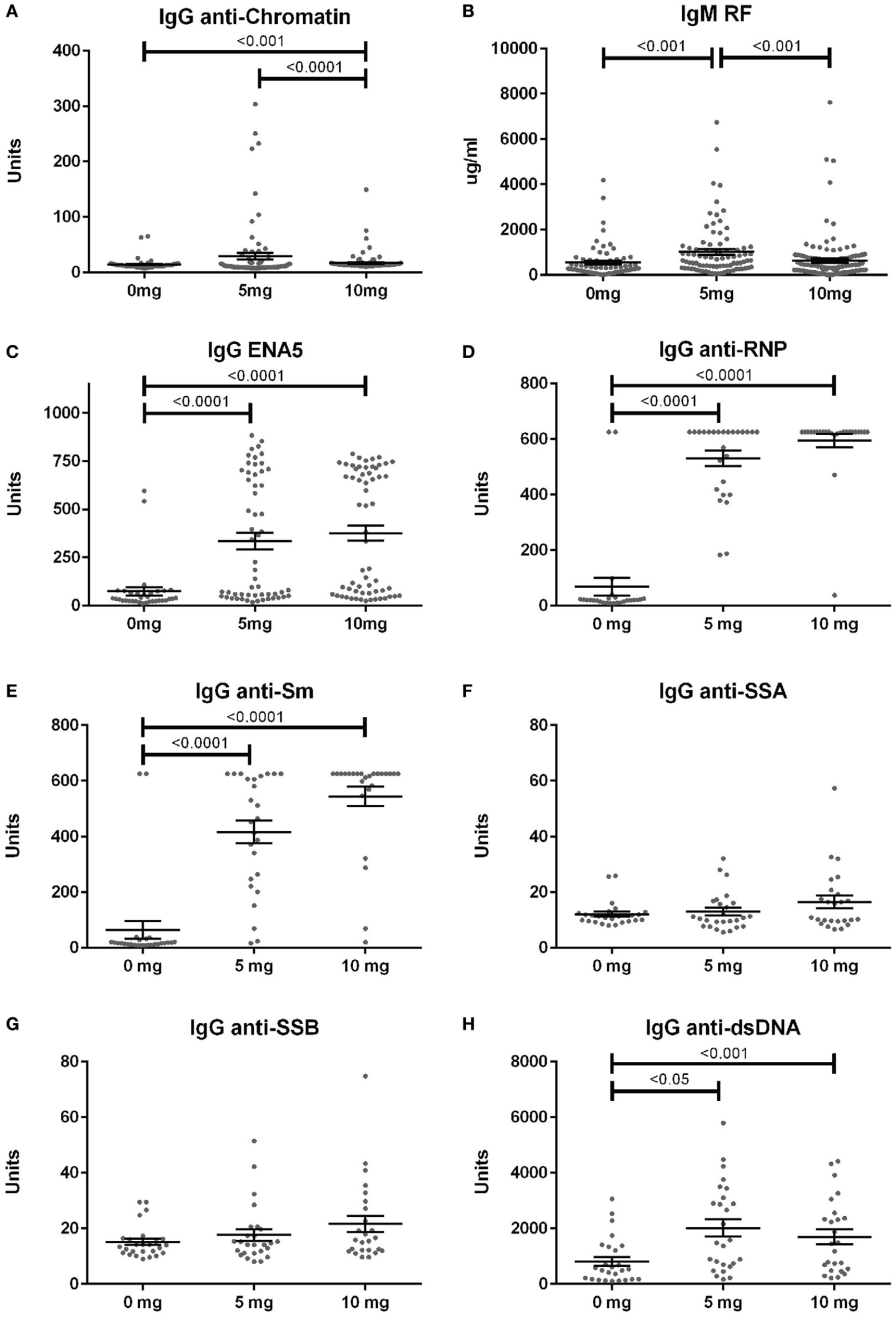
Serum autoantibodies in silica-exposed diversity outbred mice. Mice were exposed transorally to crystalline silica in PBS (see Figure 1) before blood was collected for determination of serum autoantibodies: **(A)** IgG anti-chromatin, **(B)** IgM rheumatoid factor, **(C)** ENA5 [designed for the detection of IgG anti-Sm, -RNP, -SSA (60 and 52 kDa), -SSB, and -Scl-70 IgG antibodies], **(D)** IgG anti-RNP, **(E)** IgG anti-Sm, **(F)** IgG anti-SSA, **(G)** IgG anti-SSB, and **(H)** IgG anti-dsDNA.

Anti-nuclear antibodies specificities were further examined for IgG anti-chromatin and anti-ENA5 (Sm, RNP, SS-A 60 and 52 kDa, SS-B, and Scl-70). Anti-chromatin antibodies at 12 weeks were detected in only 3% (2/68), 16% (14/86), and 4% (5/118) of mice in the 0, 5, and 10 mg groups, respectively, a much lower percentage than ANAs (Figure 4A; Table S2 in Supplementary Material). In contrast, when ANA-positive mice were examined, silica exposure was associated with substantially higher levels and percentages of anti-ENA5 antibodies compared to unexposed controls (*p* < 0.0001) (Figure 4C). Most of the ENA5 reactivity was to RNP (85 and 96% for 5 and 10 mg exposures) and Sm (58 and 84%), whereas anti-SSA (60 and 52 kDa) (12 and 20%) and anti-SSB (15 and 28%) specificities were present, but occurred less frequently (Figures 4D–G). IgM rheumatoid factor (RF) and anti-dsDNA were also elevated in a subset of silica-exposed mice with higher levels present in mice exposed to the lower dose of silica (Figures 4B,H).

In addition to the serological changes, a modest increase in spleen weight was observed in both groups of silica-exposed mice compared to controls (Figure 3C). Glomerulonephritis ranging from mild to severe was also observed by histology in a few silica-exposed mice at both doses, but in none of the PBS mice (Figure 3D). Two of the mice with glomerulonephritis developed significant proteinuria at 12 weeks (Figure 3E).

### Correlations Between Lung Pathology, Biomarkers of Silicosis, and Autoimmunity

**Figure 5:**
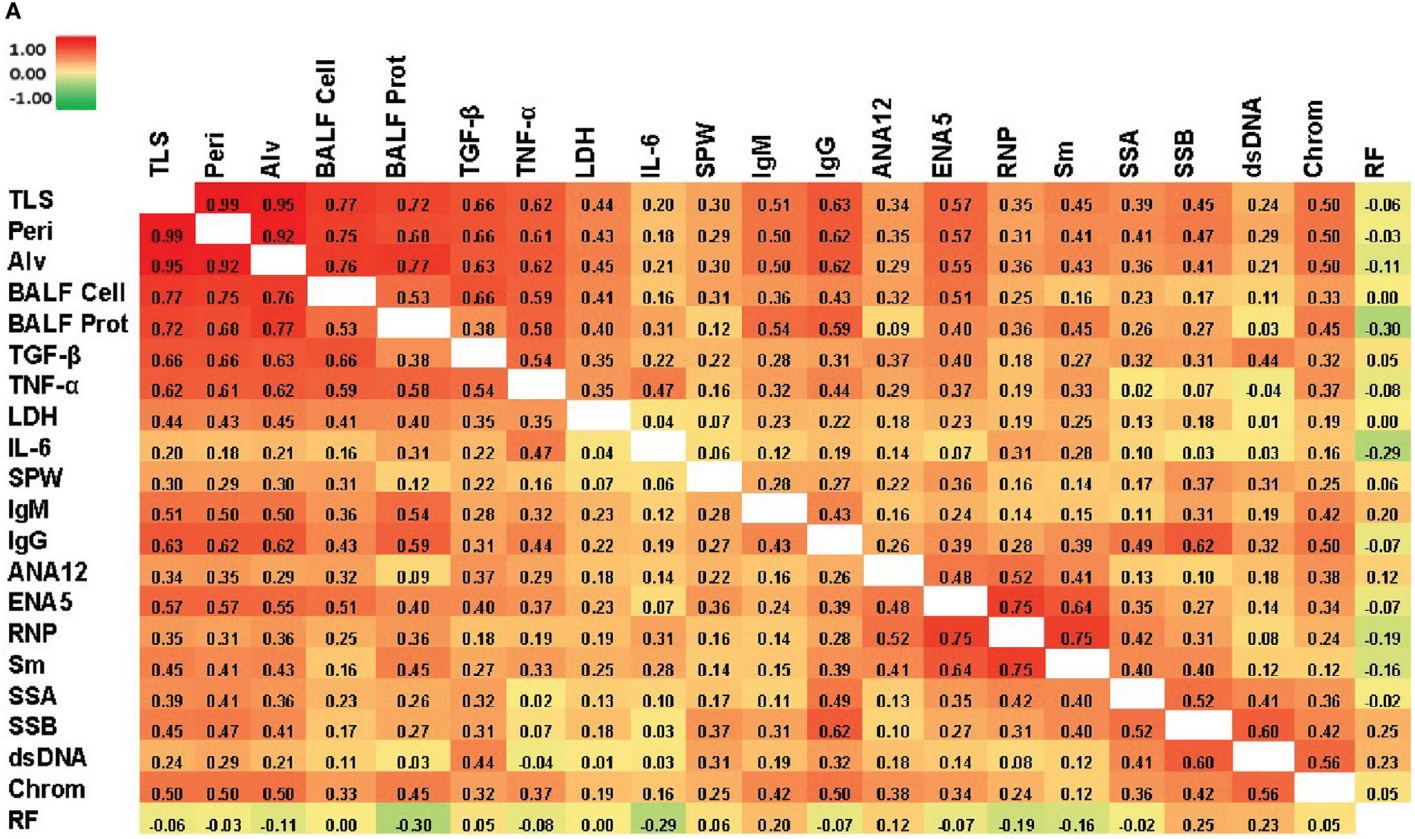

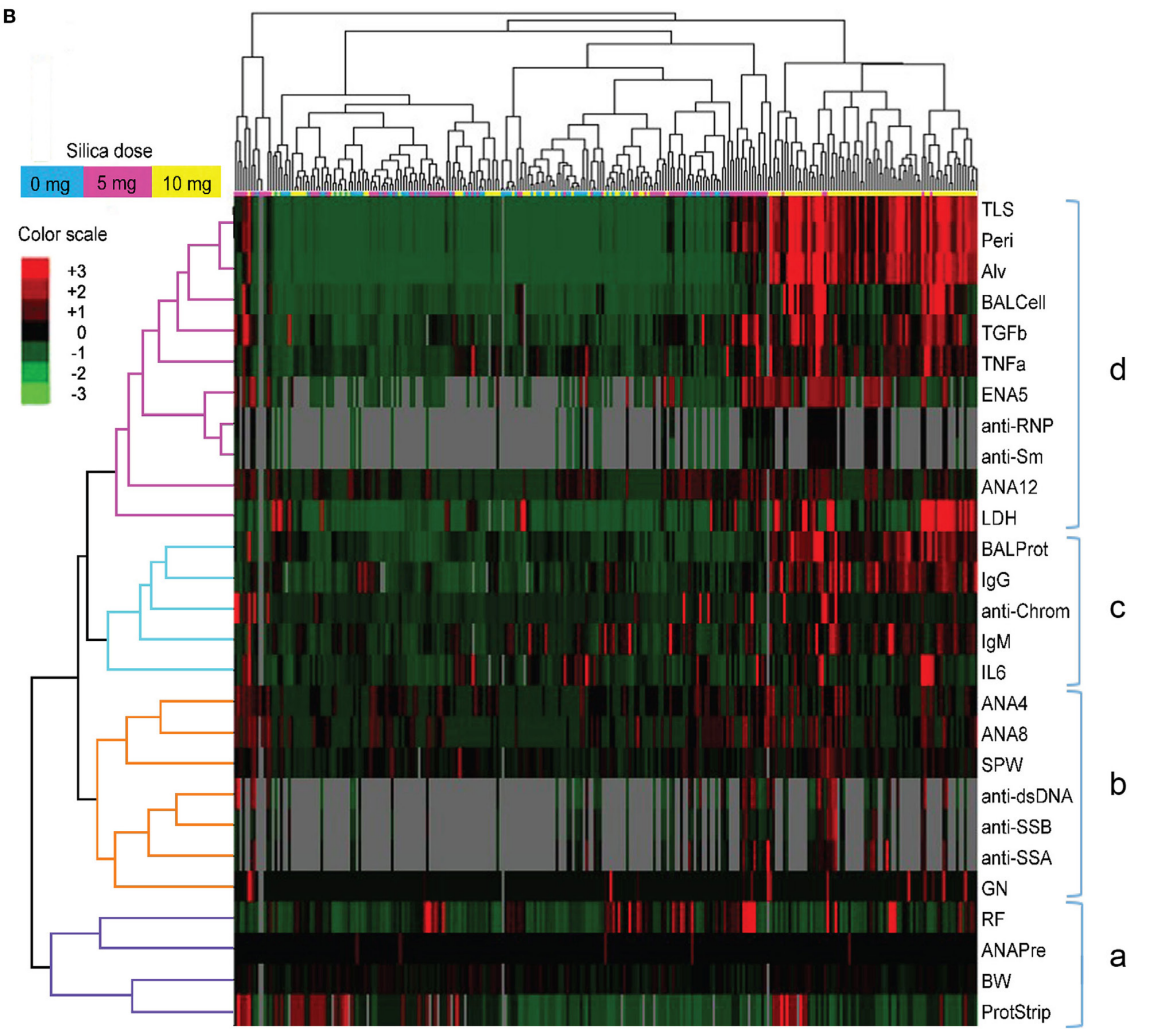
Correlations of biomarkers in silica-exposed diversity outbred mice. **(A)** Correlation matrix of Spearman’s correlation coefficient (*r_s_*) values for mice exposed transorally to 5 or 10 mg of crystalline silica. Spearman’s *r_s_* values were calculated using pairwise deletion and represented by a color gradient scale with red = 1, yellow = 0, and green = −1. **(B)** Hierarchical cluster analysis of phenotypes and samples using a complete linkage clustering method and Spearman Rank Correlation as the similarity metric. Values are reported as relative change from the average of all samples for each phenotype with red = positive relative change, black = no difference, green = negative relative change, and gray = no data available. Dose groups are indicated by the color bar below the sample cluster with blue = 0 mg, purple = 5 mg, and yellow = 10 mg. Separate clusters are defined by a, b, c, and d. Abbreviations: TLS, total lung score; Peri, perivasculitis and peribronchitis; Alv, alveolitis; BALF cell, bronchial alveolar lavage fluid (BALF) total cell number; BALF Prot, BALF protein levels; LDH, lactate dehydrogenase (LDH) activity; SPW, spleen weight; ANA12, anti-nuclear antibody (ANA) score after 12 weeks of exposure; ENA5, designed for the detection of anti-Sm, -RNP, -SS-A (60 kDa and 52 kDa), -SS-B, and -Scl-70 IgG antibodies; RNP, anti-RNP IgG; Sm, anti-Sm IgG; SSA, anti-SSA IgG; SSB, anti-SSB IgG; dsDNA, anti-dsDNA IgG; Chrom, anti-chromatin IgG, ANA4, ANA score after 4 weeks of exposure; ANA8, ANA score after 8 weeks of exposure; ANAPre, ANA score before exposure; BW, body weight; GN, glomerulonephritis; ProtStrip, urine protein using ChemStrip assay.

We next looked for correlations between the severity of silica-induced lung pathology, biomarkers of silicosis in BALF, and autoimmune disease manifestations in silica-exposed mice at 12 weeks. A significant correlation with TLS was found for all biomarkers, except IL-6, with TGF-β and TNF-α having strong and LDH activity moderate Spearman’s correlation coefficient (*r_s_*) values (Figure 5A; Table S3 in Supplementary Material). Similarly, several immune parameters, including total IgG and IgM, anti-ENA5, and anti-chromatin exhibited moderate correlation with lung pathology (Figure 5A; Table S4 in Supplementary Material). As expected, the anti-ENA5 response was strongly correlated with anti-RNP and anti-Sm, the most common ENA5 autoantibodies induced by silica (Figure 5A).

Hierarchical clustering analysis was also performed to determine unbiased relationships between biomarkers of silicosis, BALF and lung pathology, and autoimmunity, as well as between PBS and silica-exposed DO mice. Accordingly, DO mice showed clear distinction between the 10 mg dose and other groups due in large part to greater severity of lung pathology, BALF cytokines, immunoglobulin levels, and ENA5 antibodies, while there was less demarcation of the 0 and 5 mg silica groups (Figure 5B, horizontal clustering). The pulmonary and autoimmune phenotypes were divided into four main groups: RF, pre-bleed ANA, and proteinuria (cluster a); ANA, anti-dsDNA, anti-SSA, anti-SSB, and glomerulonephritis (cluster b); BALF protein and IL-6, and serum IgG, IgM, and anti-chromatin (cluster c); and lung pathology, BALF cell numbers, TGF-β, TNF-α, LDH activity, and antibodies to ENA5, Sm, and RNP (cluster d) (Figure 5B, vertical clustering). These results confirmed the connection between lung and BALF pathology and with the development of anti-ENA5, specifically anti-RNP and anti-Sm (cluster d). The separate cluster c contained mainly other immunopathologic consequences of pulmonary silicosis and inflammation. Finally, cluster b consisted of manifestations associated with systemic autoimmune diseases, such as lupus, but was more separated from silica-induced lung pathology. Thus, in DO mice certain autoimmune manifestations, such as anti-Sm and -RNP, are more strongly associated with the severity of pulmonary silicosis whereas others such as anti-dsDNA and glomerulonephritis are also dependent on other factors, possibly genetic predisposition.

### Sex Effects

**Figure 6:**
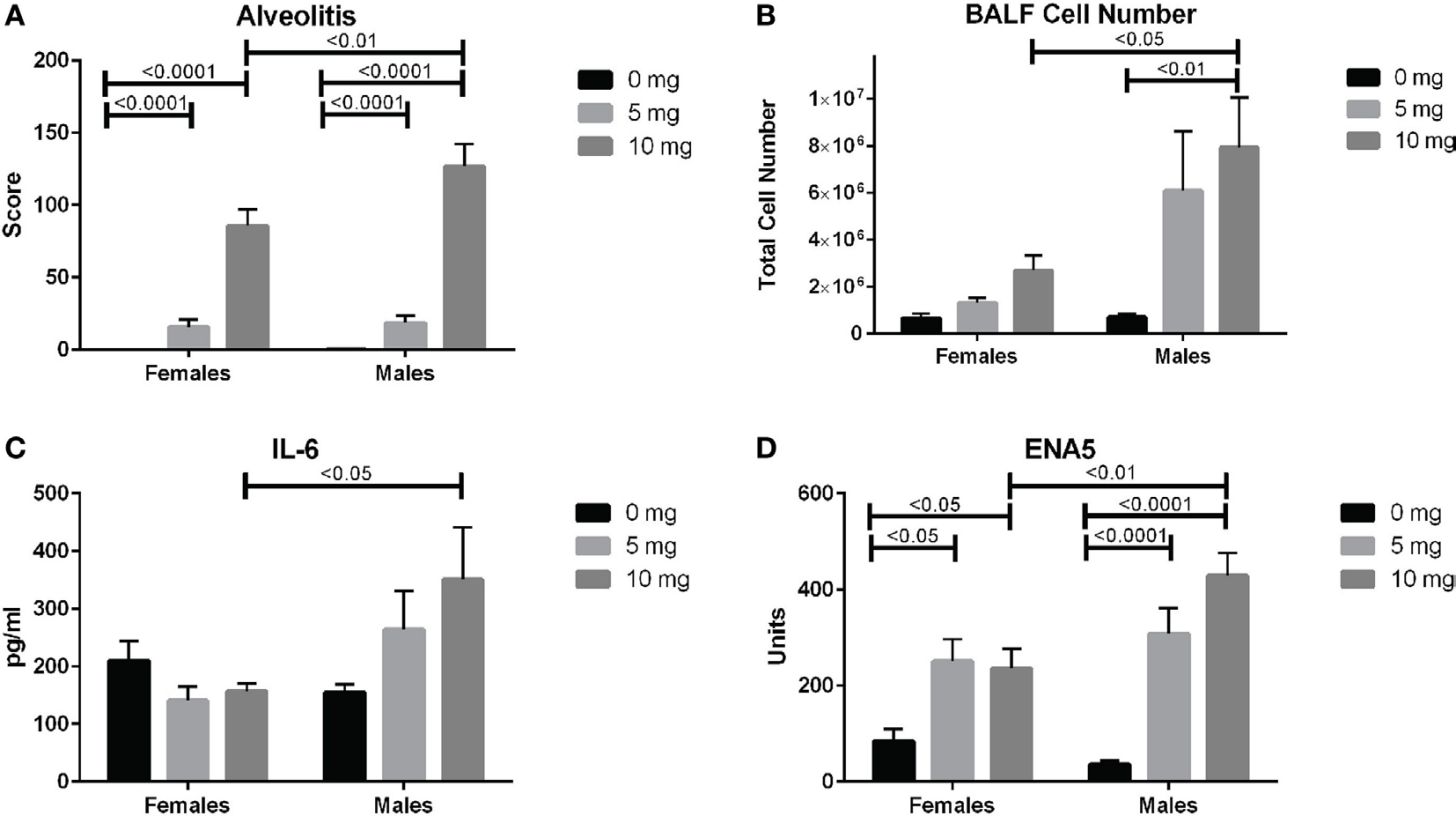
Sex differences in silica-exposed diversity outbred mice. Mice were exposed transorally to crystalline silica in PBS (see Figure 1) and females and males analyzed separately for sex effects by two-way ANOVA using Sidak’s multiple comparisons. Males exposed to 10 mg were found to have higher **(A)** alveolitis, **(B)** bronchoalveolar lavage fluid (BALF) cell number, **(C)** BALF IL-6, and **(D)** ENA5 autoantibodies compared to females at the same dose.

We next looked for potential sex effects on lung pathology, biomarkers of silicosis in BALF and autoimmunity. Using a two-way (sex and dose) ANOVA with Sidak’s multiple comparisons we found a significant sex effect in BALF cell number (*p* < 0.01), ENA5 (*p* < 0.05), urine protein levels (*p* < 0.0001), and body weight (*p* < 0.0001). Additionally, males had significantly higher alveolitis, BALF cell numbers, BALF IL-6, and ENA5 autoantibodies than females in the 10 mg dose group (Figures 6A–D). Therefore, major sex differences are reflected in greater lung inflammation, BALF cells and IL-6, and silica-induced anti-ENA5 autoantibodies in male DO mice.

## Discussion

In this study, we characterized the range of crystalline silica-associated pathology in the genetically heterogeneous DO mouse population to determine whether this model was suitable for defining causal mechanisms of both pulmonary silicosis and silica-induced autoimmunity. Indeed, a number of novel observations from this study provide strong support for that premise. First, DO mice were found to exhibit a wide variation in lung involvement, cytokine production, and importantly systemic autoimmune disease manifestations after a single inhalation exposure to silica. Second, cytokine and enzyme biomarkers associated with human pulmonary silicosis were also increased in silica-exposed mice. Third, circulating immunoglobulins and ANAs were increased with silica exposure along with the acquisition of anti-ENA specificity. Fourth, hierarchical clustering revealed that while anti-ENA5, -Sm, and -RNP responses are linked to lung pathology, other autoantibody responses (anti-DNA, -SSA, -SSB) as well as GN, are more distantly linked to the effects of silica on the lung. Additionally, about half of PBS-treated DO control mice were also ANA positive, but in contrast to silica-exposed mice, almost all lacked reactivity to common RNA and DNA antigens associated with autoimmune disease. Fifth, lupus-like glomerulonephritis although uncommon, only developed in silica-exposed mice. Finally, unlike the female bias of idiopathic systemic autoimmunity (33), sex effects of silica exposure on DO mice were more evident in males. These findings provide new insights into the effects of genetic diversity on the pathogenesis of silicosis and silica-induced autoimmunity.

In this study, a single dose of 5 and 10 mg of silica was used, which might be considered high when compared to calculated human exposures (19). However, in the absence of a standardized dose and method of administration (1, 3, 8, 16, 19, 20, 34), we selected, after comparing different routes and doses, amounts of silica in the range that produced lung pathology similar in severity to chronic human silicosis. Furthermore, the single dose by transoral instillation resulted in the most uniform presence of silica in the lung (data not shown) and reproducibility.

Silica exposure resulted in a range of lung pathologies, from mild to severe with varying degrees of peribronchial, perivascular, and alveolar inflammation even within a single dosage group. There was also a clear dose response with the 10 mg exposure group having significantly greater lung involvement and severity. These findings are consistent with previous studies in a few mouse strains that documented significant differences in the severity and type of pulmonary lesions after inhalation of silica-containing particles (9, 16, 19, 35). Less responsive strains include C57BL/6, BALB/c, and NZW while more responsive strains include C3H/HeN, MRL/MpJ, NZB, NZM2410, and (NZBxNZW)F_1_ (9, 16, 20), many of which are predisposed to lupus. Among the reported strains, only the less susceptible C57BL/6 is included in the DO founders. These findings are consistent with the variations observed in human exposures (2, 4–11) and support the importance of genetic influences on susceptibility to silicosis.

Several potential biomarkers of human silicosis identified in human exposure studies, TGF-β, TNF-α and LDH (3, 32, 36), were also elevated in the BALF of silica-exposed DO mice. Among these, TGF-β appeared to be the most robust as it was increased in both low and high dose silica exposure groups and had the strongest association with lung pathology score. In contrast, IL-6, which was only elevated in a small subset of, mainly male, DO mice exposed to the higher dose, may not be a suitable marker of silicosis, but might indicate extreme exposure. Taken together, these results are consistent with similar silica-mediated inflammatory mechanisms occurring in the lungs of humans and DO mice.

Silica-induced autoimmunity in DO mice included mild splenomegaly, autoantibodies to nuclear antigens commonly detected in lupus and mixed connective tissue disease, and glomerulonephritis. Autoimmunity was slightly more severe in the lower silica dose group, with higher levels of ANA, anti-chromatin, anti-dsDNA, RF, and glomerulonephritis than both the PBS and higher dose groups. Interestingly, and of significance in studying autoimmunity in the DO mouse, was the high prevalence (~50%) of ANAs in PBS-controls, which suggests the presence of ANA-promoting variants in many of the founder strains. Indeed, although lupus is not a major characteristic of any DO founders, lupus-predisposing loci have previously been identified in three strains, B6, 129, and NOD (37, 38). Moreover, treatment of prediabetic NOD mice with a single dose of intravenous *Mycobacterium bovis* (*M. bovis*) suppresses diabetes and induces lupus-like manifestations, including autoimmune hemolytic anemia, ANAs, and immune complex-mediated glomerulonephritis (39). Consequently, as silica exposure substantially enhances lupus in mainly susceptible strains (9, 19), the aforementioned predisposing loci responsible for spontaneous and *M. bovis*-induced lupus are also likely relevant to silica-induced autoimmunity. Thus, the DO model provides the means to identify common and unique genetic variants predisposing to spontaneous and silica-induced autoimmunity spanning a broad repertoire of laboratory and wild mouse-derived genomes.

Another striking finding was the high frequency of ENA reactivity of ANAs in both silica-exposed groups compared to PBS controls (~50 vs 6%), which included antibodies to mainly RNP and Sm, but also to SSA and SSB. In contrast, nearly all ANA positive PBS-treated mice lacked reactivity to any of the common lupus-associated autoantigens tested despite only slightly lower ANA scores than mice in the silica-exposed groups. Such DNA and ENA-negative ANAs have been associated with nonpathogenic autoantibodies such as anti-DFS70 and reduced incidence of systemic autoimmune rheumatic disease (40). Thus in DO mice, silica-induced ANAs can be distinguished from spontaneous ANAs by the presence of anti-ENA specificity, which studied in mice indicate that it is primarily mediated by TLR7 signaling (41). Similarly, anti-ENA specificities analyzed in ~1,800 uranium miners exposed to silica dust found increased prevalence of anti-SSA and anti-SSB antibodies particularly in miners with SLE or heavy exposures, however, antibodies to RNP and Sm were uncommon (42). Taken together, these findings are consistent with both genetic variation and silica exposure influencing the development of ANAs and ANA specificities.

Correlation and hierarchical clustering analyses of silica-exposed DO mice further documented the connections between lung pathology and BALF biomarkers (TGF-β, TNF-α, and LDH activity) as well as the association of these pulmonary pathologies with anti-RNP and anti-Sm. The dependence of anti-ENA5 ANAs on silica exposure is not simply secondary to inflammation, since autoimmunity induced by chronic mercury exposure leads to autoantibodies to other nuclear antigens, but not anti-ENA (43). The development of anti-ENA in nearly all ANA-positive DO mice suggests a high frequency of genetic variants predisposing to this specificity among DO founders or less likely that anti-ENA induction by silica is independent of background. In contrast, other autoimmune manifestations, such as ANA, anti-dsDNA, anti-SSA/SSB, and glomerulonephritis clustered together and were less associated with silica-induced lung pathology. These autoimmune traits are likely more dependent on genetic susceptibility than anti-ENA5 in DO mice or the responsible genetic variants are less common. Nevertheless, in either situation, our findings indicate that these autoimmune manifestations are enhanced by silica exposure similar to the early induction of severe autoimmune disease in lupus-prone strains (9, 19). Moreover, these findings argue that silica exposure in itself without the development of silicosis can contribute to autoantibody production, a finding consistent with the presence of autoantibodies in silica-exposed, but silicosis-free, humans (8). Taken together, these findings establish a new definable model to investigate genetic and pathogenic mechanisms of silica-induced autoimmunity.

Here, we document a wide variation in lung inflammatory and autoimmune disease phenotypes within the DO mouse population following inhalation of crystalline silica and also define their major associations. Consequently, our results indicate the presence of both silica-specific as well as genetically imposed autoimmune manifestations, such as anti-ENA and ANA positivity. Thus, exposure of the DO mouse to silica provides a model of the diverse pathologic and autoimmune responses of the human population to environmental silica. Accordingly, the DO system provides a unique opportunity to investigate the genetic pathways and pathophysiologic mechanisms critical for promoting silica-associated autoimmune disease and supports the application of this model in investigating population wide environmental effects.

## Data Availability Statement

The dataset analyzed for this study can be found in the supplementary materials for this article.

## Ethics Statement

This study was carried out in accordance with the recommendations of The Scripps Research Institute Institutional Animal Care and Use Committee (IACUC). The protocol was approved by The Scripps Research Institute IACUC.

## Author Contributions

JM, DK, and KP contributed to conception and design of the study. JM, GE, JC, and RP contributed to mouse maintenance, sample acquisition, execution of experiments, and data analysis. JM, DK, and KP wrote the first draft of the manuscript. All authors contributed to manuscript revision, read and approved the submitted version.

## Conflict of Interest Statement

The authors declare that the research was conducted in the absence of any commercial or financial relationships that could be construed as a potential conflict of interest.

## Footnotes

^1^http://bonsai.hgc.jp/~mdehoon/software/cluster/software.htm (Accessed: July 6, 2017).

^2^http://jtreeview.sourceforge.net/ (Accessed: July 6, 2017).

## References

[1] Leung CC Yu IT Chen W. Silicosis. Lancet (2012) 379(9830):2008–18. 10.1016/S0140-6736(12)60235-9 22534002

[2] Pollard KM Silica, silicosis, and autoimmunity. Front Immunol (2016) 7:97 10.3389/fimmu.2016.00097 27014276 PMC4786551

[3] Kawasaki H. A mechanistic review of silica-induced inhalation toxicity. Inhal Toxicol (2015) 27(8):363–77. 10.3109/08958378.2015.1066905 26194035

[4] Steenland K Brown D. Mortality study of gold miners exposed to silica and nonasbestiform amphibole minerals: an update with 14 more years of follow-up. Am J Ind Med (1995) 27(2):217–29. 10.1002/ajim.4700270207 7755012

[5] Wilke RA Salisbury S Abdel-Rahman E Brazy PC Lupus-like autoimmune disease associated with silicosis. Nephrol Dial Transplant (1996) 11(9):1835–8. 10.1093/ndt/11.9.1835 8918632

[6] Brown LM Gridley G Olsen JH Mellemkjaer L Linet MS Fraumeni JF Jr. Cancer risk and mortality patterns among silicotic men in Sweden and Denmark. J Occup Environ Med (1997) 39(7):633–8. 10.1097/00043764-199707000-00008 9253724

[7] Haustein UF Anderegg U Silica induced scleroderma – clinical and experimental aspects. J Rheumatol (1998) 25(10):1917–26. 9779844

[8] Parks CG Conrad K Cooper GS Occupational exposure to crystalline silica and autoimmune disease. Environ Health Perspect (1999) 107(Suppl 5):793–802. 10.2307/3434342 10970168 PMC1566238

[9] Brown JM Archer AJ Pfau JC Holian A. Silica accelerated systemic autoimmune disease in lupus-prone New Zealand mixed mice. Clin Exp Immunol (2003) 131(3):415–21. 10.1046/j.1365-2249.2003.02094.x 12605693 PMC1808650

[10] Steenland K. One agent, many diseases: exposure-response data and comparative risks of different outcomes following silica exposure. Am J Ind Med (2005) 48(1):16–23. 10.1002/ajim.20181 15940719

[11] Miller FW Alfredsson L Costenbader KH Kamen DL Nelson LM Norris JM Epidemiology of environmental exposures and human autoimmune diseases: findings from a national institute of environmental health sciences expert panel workshop. J Autoimmun (2012) 39(4):259–71. 10.1016/j.jaut.2012.05.002 22739348 PMC3496812

[12] Pollard KM. Gender differences in autoimmunity associated with exposure to environmental factors. J Autoimmun (2012) 38(2–3):J177–86. 10.1016/j.jaut.2011.11.007 22137891 PMC3302961

[13] Doll NJ Stankus RP Hughes J Weill H Gupta RC Rodriguez M Immune complexes and autoantibodies in silicosis. J Allergy Clin Immunol (1981) 68(4):281–5. 10.1016/0091-6749(81)90152-4 6974746

[14] Conrad K Stahnke G Liedvogel B Mehlhorn J Barth J Blasum C Anti-CENP-B response in sera of uranium miners exposed to quartz dust and patients with possible development of systemic sclerosis (scleroderma). J Rheumatol (1995) 22(7):1286–94. 7562760

[15] Conrad K Mehlhorn J Luthke K Dorner T Frank KH. Systemic lupus erythematosus after heavy exposure to quartz dust in uranium mines: clinical and serological characteristics. Lupus (1996) 5(1):62–9. 10.1177/096120339600500112 8646229

[16] Davis GS Leslie KO Hemenway DR. Silicosis in mice: effects of dose, time, and genetic strain. J Environ Pathol Toxicol Oncol (1998) 17(2):81–97. 9546745

[17] Pfau JC Brown JM Holian A. Silica-exposed mice generate autoantibodies to apoptotic cells. Toxicology (2004) 195(2–3):167–76. 10.1016/j.tox.2003.09.011 14751672

[18] Germolec D Kono DH Pfau JC Pollard KM. Animal models used to examine the role of the environment in the development of autoimmune disease: findings from an NIEHS expert panel workshop. J Autoimmun (2012) 39(4):285–93. 10.1016/j.jaut.2012.05.020 22748431 PMC3465484

[19] Bates MA Brandenberger C Langohr I Kumagai K Harkema JR Holian A Silica triggers inflammation and ectopic lymphoid neogenesis in the lungs in parallel with accelerated onset of systemic autoimmunity and glomerulonephritis in the lupus-prone NZBWF1 mouse. PLoS One (2015) 10(5):e0125481. 10.1371/journal.pone.0125481 25978333 PMC4433215

[20] Callis AH Sohnle PG Mandel GS Wiessner J Mandel NS. Kinetics of inflammatory and fibrotic pulmonary changes in a murine model of silicosis. J Lab Clin Med (1985) 105(5):547–53. 2985721

[21] Parker CC Palmer AA. Dark matter: are mice the solution to missing heritability? Front Genet (2011) 2:32. 10.3389/fgene.2011.00032 22303328 PMC3268586

[22] Churchill GA Gatti DM Munger SC Svenson KL. The diversity outbred mouse population. Mamm Genome (2012) 23(9–10):713–8. 10.1007/s00335-012-9414-2 22892839 PMC3524832

[23] Svenson KL Gatti DM Valdar W Welsh CE Cheng R Chesler EJ High-resolution genetic mapping using the mouse diversity outbred population. Genetics (2012) 190(2):437–47. 10.1534/genetics.111.132597 22345611 PMC3276626

[24] Logan RW Robledo RF Recla JM Philip VM Bubier JA Jay JJ High-precision genetic mapping of behavioral traits in the diversity outbred mouse population. Genes Brain Behav (2013) 12(4):424–37. 10.1111/gbb.12029 23433259 PMC3709837

[25] Kelly SA Pomp D. Genetic determinants of voluntary exercise. Trends Genet (2013) 29(6):348–57. 10.1016/j.tig.2012.12.007 23351966 PMC3665695

[26] Church RJ Gatti DM Urban TJ Long N Yang X Shi Q Sensitivity to hepatotoxicity due to epigallocatechin gallate is affected by genetic background in diversity outbred mice. Food Chem Toxicol (2015) 76:19–26. 10.1016/j.fct.2014.11.008 25446466 PMC4324012

[27] French JE Gatti DM Morgan DL Kissling GE Shockley KR Knudsen GA Diversity outbred mice identify population-based exposure thresholds and genetic factors that influence benzene-induced genotoxicity. Environ Health Perspect (2015) 123(3):237–45. 10.1289/ehp.1408202 25376053 PMC4348743

[28] Biswas R Trout KL Jessop F Harkema JR Holian A. Imipramine blocks acute silicosis in a mouse model. Part Fibre Toxicol (2017) 14(1):36. 10.1186/s12989-017-0217-1 28893276 PMC5594487

[29] Hamilton RF Jr Thakur SA Mayfair JK Holian A. MARCO mediates silica uptake and toxicity in alveolar macrophages from C57BL/6 mice. J Biol Chem (2006) 281(45):34218–26. 10.1074/jbc.M605229200 16984918

[30] Toomey CB Cauvi DM Hamel JC Ramirez AE Pollard KM. Cathepsin B regulates the appearance and severity of mercury-induced inflammation and autoimmunity. Toxicol Sci (2014) 142(2):339–49. 10.1093/toxsci/kfu189 25237059 PMC4250846

[31] Koh YT Scatizzi JC Gahan JD Lawson BR Baccala R Pollard KM Role of nucleic acid-sensing TLRs in diverse autoantibody specificities and anti-nuclear antibody-producing B cells. J Immunol (2013) 190(10):4982–90. 10.4049/jimmunol.1202986 23589617 PMC3729324

[32] Gulumian M Borm PJ Vallyathan V Castranova V Donaldson K Nelson G Mechanistically identified suitable biomarkers of exposure, effect, and susceptibility for silicosis and coal-worker’s pneumoconiosis: a comprehensive review. J Toxicol Environ Health B Crit Rev (2006) 9(5):357–95. 10.1080/15287390500196537 16990219

[33] Pollard KM Hultman P Toomey CB Cauvi DM Hoffman HM Hamel JC Definition of IFN-gamma-related pathways critical for chemically-induced systemic autoimmunity. J Autoimmun (2012) 39(4):323–31. 10.1016/j.jaut.2012.04.003 22578563 PMC3570757

[34] Barbarin V Nihoul A Misson P Arras M Delos M Leclercq I The role of pro- and anti-inflammatory responses in silica-induced lung fibrosis. Respir Res (2005) 6:112. 10.1186/1465-9921-6-112 16212659 PMC1274346

[35] Bates MA Brandenberger C Langohr II Kumagai K Lock AL Harkema JR Silica-triggered autoimmunity in lupus-prone mice blocked by docosahexaenoic acid consumption. PLoS One (2016) 11(8):e0160622. 10.1371/journal.pone.0160622 27513935 PMC4981380

[36] Vanhee D Gosset P Boitelle A Wallaert B Tonnel AB. Cytokines and cytokine network in silicosis and coal workers’ pneumoconiosis. Eur Respir J (1995) 8(5):834–42. 7656959

[37] Jordan MA Silveira PA Shepherd DP Chu C Kinder SJ Chen J Linkage analysis of systemic lupus erythematosus induced in diabetes-prone nonobese diabetic mice by *Mycobacterium bovis*. J Immunol (2000) 165(3):1673–84. 10.4049/jimmunol.165.3.1673 10903779

[38] Carlucci F Cortes-Hernandez J Fossati-Jimack L Bygrave AE Walport MJ Vyse TJ Genetic dissection of spontaneous autoimmunity driven by 129-derived chromosome 1 loci when expressed on C57BL/6 mice. J Immunol (2007) 178(4):2352–60. 10.4049/jimmunol.178.4.2352 17277141

[39] Baxter AG Horsfall AC Healey D Ozegbe P Day S Williams DG Mycobacteria precipitate an SLE-like syndrome in diabetes-prone NOD mice. Immunology (1994) 83(2):227–31. 7835939 PMC1414944

[40] Gundin S Irure-Ventura J Asensio E Ramos D Mahler M Martinez-Taboada V Measurement of anti-DFS70 antibodies in patients with ANA-associated autoimmune rheumatic diseases suspicion is cost-effective. Auto Immun Highlights (2016) 7(1):10. 10.1007/s13317-016-0082-1 27473142 PMC4967047

[41] Kono DH Baccala R Theofilopoulos AN. TLRs and interferons: a central paradigm in autoimmunity. Curr Opin Immunol (2013) 25(6):720–7. 10.1016/j.coi.2013.10.006 24246388 PMC4309276

[42] Conrad K Mehlhorn J. Diagnostic and prognostic relevance of autoantibodies in uranium miners. Int Arch Allergy Immunol (2000) 123(1):77–91. 10.1159/000024426 11014974

[43] Hultman P Bell LJ Enestrom S Pollard KM. Murine susceptibility to mercury. I. Autoantibody profiles and systemic immune deposits in inbred, congenic, and intra-H-2 recombinant strains. Clin Immunol Immunopathol (1992) 65(2):98–109. 10.1016/0090-1229(92)90212-7 1395135

