# Supplementary material for: Silicosis and Silica-Induced Autoimmunity in the Diversity Outbred Mouse
Source: Front Immunol. 2018 Apr 26;9:874. doi: 10.3389/fimmu.2018.00874 (PMC5932595; doi:10.3389/fimmu.2018.00874)
Supplement: Supplementary file 1 [file data_sheet_1.pdf]

Supplementary Material

Silicosis and silica-induced autoimmunity in the diversity outbred mouse

Jessica M. Mayeux[1], Gabriela M. Escalante[1], Joseph M. Christy[1], Rahul D. Pawar[1], Dwight H. Kono[2], and Kenneth M. Pollard[1*]

[1]Department of Molecular Medicine1, [2]Department of Immunology and Microbiology, The Scripps Research Institute, 10550 North Torrey Pines Road, La Jolla, CA 92037, USA

*** Correspondence:**
K. M. Pollard, PhD, Department of Molecular Medicine, MEM-125, The Scripps Research Institute, 10550 North Torrey Pines Road, La Jolla, CA 92037, USA. Tel: 858-784-9214, Fax 858 784-8836, Email mpollard@scripps.edu

Table S1. Silicosis disease descriptive statistics in DO mice

	0 mg		5 mg		10 mg	
	mean±SD	n (%)	mean±SD	n (%)	mean±SD	n (%)
Total Lung Score	2.0±7.6	2/67 (3.0)	65.5±100	36/86 (41.9)	236±224	78/118 (66.1)
Alveolitis	0.17±0.89	1/67 (1.5)	17.0±33.9	40/86 (46.5)	1069±105	81/118 (68.6)
Perivasculitis and Peribronchitis	1.8±6.83	2/67 (3.0)	48.6±71.3	37/86 (43.0)	130±123	79/118 (66.9)
BALF Cell Number (total)	0.68±0.93	2/67 (3.0)	3.6±11.7	15/86 (17.4)	5.3±12.2	46/117 (39.3)
BALF Protein	217±141	1/68 (1.5)	150±108	1/86 (1.2)	709±669	63/118 (53.4)
LDH activity	1.9±3.8	4/67 (6.0)	3.4±6.3	8/86 (9.3)	8.0±13.1	30/117 (25.6)
TNF-α	25.3±24.0	2/67 (3.0)	42.4±117	6/86 (7.0)	50.1±39.7	23/116 (19.8)
TGF-β	27.4±60.3	1/66 (1.5)	83.2±78.3	11/86 (12.8)	84.9±84.0	24/116 (20.7)
IL-6	183±151	4/67 (6.0)	201±332	6/86 (7.0)	254±498	6/116 (5.2)

Male and female 8-10-week non-sibling DO mice (n = 280) were exposed to a single 0 mg (n=70), 5 mg (n=90), or 10 mg (n=120) dose of transoral crystalline silica in PBS. After 12 weeks, lung pathology was scored and BALF was measured for cytokines and other silicosis biomarkers. Mean±standard deviation (SD) are reported in one column while the affected/total numbers (n) and percent (%) in parenthesis are in the second column for each dose. The percent positive cutoff was defined as the mean plus two SD of the control 0 mg group for each phenotype. Abbreviations: BALF, bronchial alveolar lavage fluid; LDH, lactate dehydrogenase.

Table S2. Autoimmune disease descriptive statistics in DO mice

	0 mg		5 mg		10 mg	
	mean±SD	n (%)	mean±SD	n (%)	mean±SD	n (%)
IgG	1928±1573	2/66 (3.0)	1800±1298	3/84 (3.6)	4604±4121	45/116 (38.8)
IgM	318±268	3/68 (4.4)	247±228	1/85 (1.2)	459±299	12/118 (10.2)
ANA	1.2±1.4	34/68 (50.0)	1.8±1.6	57/86 (66.3)	1.3±1.4	63/118 (53.4)
IgG anti-Chromatin	14.0±9.4	2/68 (2.9)	28.8±54.4	14/86 (16.3)	17.2±14.9	5/118 (4.2)
IgM RF	558±734	3/67 (4.5)	1021±1167	12/86 (14.0)	639±1037	6/118 (5.1)
ENA5	72.9±128	2/34 (5.9)	334±316	26/56 (46.4)	376±311	31/61 (50.8)
IgG anti-RNP	68.7±165	2/26 (7.7)	531±138	22/26 (84.6)	594±120	24/25 (96.0)
IgG anti-Sm	64.2±165.4	2/26 (7.7)	416±208	15/26 (57.7)	544±174	21/25 (84.0)
IgG anti-SSA	12.1±4.4	2/26 (7.7)	13.0±6.9	3/26 (11.5)	16.5±11.3	5/25 (20.0)
IgG anti-SSB	15.2±5.9	2/26 (7.7)	17.6±10.4	4/26 (15.4)	21.7±14.8	7/25 (28.0)
IgG anti-dsDNA	817±822	2/26 (7.7)	2021±1562	11/26 (42.3)	1696±1325	6/25 (24.0)
Spleen Weight	0.08±0.03	1/67 (1.5)	0.1±0.04	9/85 (10.6)	0.1±0.04	6/118 (5.1)
Glomerulonephritis	0.0±0.0	0/67 (0.0)	0.15±0.47	10/87 (11.5)	0.16±0.65	8/118 (6.8)
Proteinuria	35.5±68.0	1/62 (1.6)	33.6±60.8	1/81 (1.2)	50.4±101	5/113 (4.4)

Mice and exposure to silica as in Table S1. After 12 weeks exposure kidney pathology was scored and serum immunoglobulins and autoantibodies were quantified. Mean±standard deviation (SD) are reported in one column while the affected/total numbers (n) and percent (%) in parenthesis are in the second column for each dose. The percent positive cutoff was defined as the mean plus two SD of the control 0 mg group for each phenotype except ANA which was considered positive for ANA>0. ENA5 was run only on ANA^+ mice. RNP, Sm, SSA, SSB, and dsDNA were run on a subset of $ENA5^+$ mice. Abbreviations: ANA, anti-nuclear antibody; ENA5, designed for the detection of anti-Sm, -RNP, -SS-A (60kDa and 52kDa), -SS-B and -Scl-70 IgG; Proteinuria; urine protein using Chemstrip assay.

Table S3. Silicosis disease correlations in DO mice

	Correlation (r_s)	Strength of r_s	95% CI	Sample Size	P-value of r_s
Correlation of Total Lung Score with lung features					
Perivasculitis and Peribronchitis	0.99	Very Strong	0.99 to 0.99	204	< 0.0001
Alveolitis	0.95	Very Strong	0.94 to 0.96	204	< 0.0001
BALF Cell Number	0.77	Strong	0.71 to 0.82	203	< 0.0001
BALF Protein	0.72	Strong	0.64 to 0.78	204	< 0.0001
TGF-β	0.66	Strong	0.57 to 0.73	202	< 0.0001
TNF-α	0.62	Strong	0.52 to 0.70	202	< 0.0001
LDH Activity	0.44	Moderate	0.32 to 0.55	203	< 0.0001
IL-6	0.20	Weak	0.06 to 0.33	202	0.0050
Body Weight	0.04	Very Weak	-0.1 to 0.18	204	0.5341
Correlation of TGF-β in BALF with lung features					
Total Lung Score	0.66	Strong	0.57 to 0.73	202	< 0.0001
Perivasculitis and Peribronchitis	0.66	Strong	0.57 to 0.73	202	< 0.0001
Alveolitis	0.63	Strong	0.53 to 0.71	202	< 0.0001
BALF Cell Number	066	Strong	0.57 to 0.73	202	< 0.0001
BALF Protein	0.38	Weak	0.25 to 0.48	202	< 0.0001
TNF-α	0.54	Moderate	0.43 to 0.63	202	< 0.0001
LDH Activity	0.35	Weak	0.21 to 0.47	202	< 0.0001
IL-6	0.22	Very Weak	0.08 to 0.35	202	0.0019
Body Weight	-0.13	Very Weak	-0.26 to 0.02	202	0.0741
Correlation of TNF-α in BALF with lung features					
Total Lung Score	0.62	Strong	0.52 to 0.70	202	< 0.0001
Perivasculitis and Peribronchitis	0.61	Strong	0.51 to 0.69	202	< 0.0001
Alveolitis	0.62	Strong	0.53 to 0.70	202	< 0.0001
BALF Cell Number	0.59	Moderate	0.49 to 0.68	202	< 0.0001
BALF Protein	0.58	Moderate	0.48 to 0.67	202	< 0.0001
TGF-β	0.54	Moderate	0.43 to 0.63	202	< 0.0001
LDH Activity	0.34	Weak	0.22 to 0.47	202	< 0.0001
IL-6	0.47	Moderate	0.35 to 0.57	202	< 0.0001
Body Weight	0.10	Very Weak	-0.04 to 0.24	202	0.1398
Correlation of LDH activity in BALF with lung features					
Total Lung Score	0.44	Moderate	0.32 to 0.55	203	< 0.0001
Perivasculitis and Peribronchitis	0.43	Moderate	0.31 to 0.54	203	< 0.0001
Alveolitis	0.45	Moderate	0.33 to 0.56	203	< 0.0001
BALF Cell Number	0.41	Moderate	0.29 to 0.53	202	< 0.0001
BALF Protein	0.40	Moderate	0.28 to 0.52	203	< 0.0001
TGF-β	0.35	Weak	0.21 to 0.47	202	< 0.0001

TNF-α	0.35	Weak	0.22 to 0.47	202	< 0.0001
IL-6	0.04	Very Weak	-0.10 to 0.18	202	0.5383
Body Weight	-0.03	Very Weak	-0.17 to 0.11	203	0.6926
Correlation of IL-6 in BALF with lung features					
Total Lung Score	0.20	Weak	0.06 to 0.33	202	0.0050
Perivasculitis and Peribronchitis	0.18	Very Weak	0.04 to 0.32	202	0.0099
Alveolitis	0.21	Weak	0.07 to 0.34	202	0.0024
BALF Cell Number	0.16	Very Weak	0.02 to 0.30	202	0.0216
BALF Protein	0.31	Weak	0.17 to 0.43	202	< 0.0001
TGF-β	0.22	Weak	0.08 to 0.35	202	0.0019
TNF-α	0.47	Moderate	0.35 to 0.57	202	< 0.0001
LDH Activity	0.04	Very Weak	-0.10 to 0.18	202	0.5383
Body Weight	-0.03	Very Weak	0.00 to 0.27	202	0.0501

Mice and exposure to silica as in Table S1. After 12 weeks exposure, lung pathology was scored and BALF quantified for cytokines and other silicosis biomarkers. Spearman correlation coefficients (r_s) were calculated and interpreted as very weak (0-0.19), weak (0.20-0.39), moderate (0.40-0.59), strong (0.60-0.79), or very strong (0.8-1.0). The 95% confidence interval (95% CI), sample size, and P-values are shown.

Table S4. Autoimmune disease correlations in DO mice

	Correlation (r_s)	Strength of r_s	95% CI	Sample Size	P-value of r_s
Correlation of serum IgG with lung features					
Total Lung Score	0.63	Strong	0.54 to 0.71	200	< 0.0001
Perivasculitis and Peribronchitis	0.62	Strong	0.53 to 0.70	200	< 0.0001
Alveolitis	0.62	Strong	0.53 to 0.70	200	< 0.0001
BALF Cell Number	0.43	Moderate	0.30 to 0.54	199	< 0.0001
BALF Protein	0.59	Moderate	0.49 to 0.68	200	< 0.0001
TGF-β	0.31	Weak	0.17 to 0.43	198	< 0.0001
TNF-α	0.44	Moderate	0.31 to 0.54	198	< 0.0001
LDH Activity	0.22	Weak	0.07 to 0.35	199	0.0022
IL-6	0.19	Very Weak	0.05 to 0.33	198	0.0063
Body Weight	-0.13	Very Weak	-0.27 to 0.01	204	0.0612
Correlation of serum IgM with lung features					
Total Lung Score	0.51	Moderate	0.40 to 0.61	203	< 0.0001
Perivasculitis and Peribronchitis	0.50	Moderate	0.39 to 0.60	203	< 0.0001
Alveolitis	0.50	Moderate	0.39 to 0.60	203	< 0.0001
BALF Cell Number	0.36	Weak	0.23 to 0.48	202	< 0.0001
BALF Protein	0.54	Moderate	0.44 to 0.64	203	< 0.0001
TGF-β	0.28	Weak	0.14 to 0.40	201	< 0.0001
TNF-α	0.32	Weak	0.19 to 0.45	201	< 0.0001
LDH Activity	0.23	Weak	0.09 to 0.36	202	0.0013
IL-6	0.12	Very Weak	-0.03 to 0.25	201	0.1016
Body Weight	-0.02	Very Weak	-0.17 to 0.12	203	0.7322
Correlation of serum ANA with lung features					
Total Lung Score	0.34	Weak	0.21 to 0.46	204	< 0.0001
Perivasculitis and Peribronchitis	0.35	Weak	0.22 to 0.47	204	< 0.0001
Alveolitis	0.29	Weak	0.16 to 0.42	204	< 0.0001
BALF Cell Number	0.32	Weak	0.18 to 0.44	203	< 0.0001
BALF Protein	0.09	Very Weak	-0.05 to 0.23	204	0.1948
TGF-β	0.37	Weak	0.24 to 0.48	202	< 0.0001
TNF-α	0.29	Weak	0.16 to 0.42	202	< 0.0001
LDH Activity	0.18	Very Weak	0.04 to 0.31	203	0.0115
IL-6	0.14	Very Weak	0.00 to 0.28	200	0.0398
Body Weight	-0.02	Very Weak	-0.16 to 0.12	204	0.8071
Correlation of serum IgG anti-ENA5 with lung features					
Total Lung Score	0.57	Moderate	0.45 to 0.67	163	< 0.0001
Perivasculitis and Peribronchitis	0.57	Moderate	0.45 to 0.67	163	< 0.0001

Alveolitis	0.55	Moderate	0.43 to 0.65	163	< 0.0001
BALF Cell Number	0.51	Moderate	0.39 to 0.62	162	< 0.0001
BALF Protein	0.40	Moderate	0.26 to 0.53	163	< 0.0001
TGF-β	0.40	Moderate	0.26 to 0.53	161	< 0.0001
TNF-α	0.37	Weak	0.23 to 0.50	161	< 0.0001
LDH Activity	0.23	Weak	0.07 to 0.38	162	0.0030
IL-6	0.07	Very Weak	-0.09 to 0.23	161	0.3645
Body Weight	0.07	Very Weak	-0.09 to 0.22	163	0.4092
Correlation of serum IgG anti-chromatin with lung features					
Total Lung Score	0.50	Moderate	0.39 to 0.60	204	< 0.0001
Perivasculitis and Peribronchitis	0.50	Moderate	0.38 to 0.60	204	< 0.0001
Alveolitis	0.50	Moderate	0.39 to 0.60	204	< 0.0001
BALF Cell Number	0.33	Weak	0.20 to 0.45	203	< 0.0001
BALF Protein	0.45	Moderate	0.33 to 0.56	204	< 0.0001
TGF-β	0.32	Weak	0.18 to 0.44	202	< 0.0001
TNF-α	0.37	Weak	0.25 to 0.49	202	< 0.0001
LDH Activity	0.19	Very Weak	0.05 to 0.32	203	0.0072
IL-6	0.16	Very Weak	0.02 to 0.30	202	0.0212
Body Weight	-0.11	Very Weak	-0.25 to 0.03	204	0.1051
Correlation of serum IgM Rheumatoid Factor with lung features					
Total Lung Score	-0.06	Very Weak	-0.20 to 0.08	204	0.4103
Perivasculitis and Peribronchitis	-0.03	Very Weak	-0.17 to 0.11	204	0.6470
Alveolitis	-0.11	Very Weak	-0.25 to 0.03	204	0.1134
BALF Cell Number	0.00	Very Weak	-0.15 to 0.14	203	0.9571
BALF Protein	-0.30	Weak	-0.42 to -0.16	204	< 0.0001
TGF-β	0.05	Very Weak	-0.10 to 0.19	202	0.5018
TNF-α	-0.08	Very Weak	-0.22 to 0.06	202	0.2570
LDH Activity	0.00	Very Weak	-0.14 to 0.14	203	0.9808
IL-6	-0.29	Weak	-0.41 to -0.15	202	< 0.0001
Body Weight	-0.31	Weak	-0.44 to -0.18	204	< 0.0001

Mice and silica exposure as in Table S1. Lung pathology scores, BALF analysis, and serological measurements as in Tables S1 and S2. Spearman correlation coefficients (r_s) were calculated and interpreted as very weak (0-0.19), weak (0.20-0.39), moderate (0.40-0.59), strong (0.60-0.79), or very strong (0.8-1.0). The 95% confidence interval (95% CI), sample size, and P-values are shown.
